# SGIV envelope protein VP088 facilitated virus replication via interacting with other viral proteins and promoting p62-dependent autophagic degradation of TBK1

**DOI:** 10.1128/jvi.01193-25

**Published:** 2025-12-11

**Authors:** Mengdi Yuan, Ya Zhang, Xiaolin Gao, Wenji Wang, Yin Zhao, Qiwei Qin, Xiaohong Huang, Youhua Huang

**Affiliations:** 1 College of Marine Sciences, South China Agricultural University 12526, Guangzhou, China; 2 Nansha-South China Agricultural University Fishery Research Institute 12526, Guangzhou, China; 3 Southern Marine Science and Engineering Guangdong Laboratory 590852 , Zhuhai, China; Michigan State University, East Lansing, Michigan, USA

**Keywords:** iridovirus, envelope protein, VP088, TBK1, IFN, autophagic degradation

## Abstract

Singapore grouper iridovirus (SGIV), a novel member of the genus *Ranavirus*, family Iridoviridae, frequently causes a severe disease with high mortality in grouper aquaculture. Although previous findings have demonstrated that SGIV envelope protein VP088 was crucial for its infectivity, the underlying mechanism still remained uncertain. Here, we screened the potential viral proteins that interacted with VP088 during SGIV infection using GFP pull-down assay. Co-immunoprecipitation (Co-IP) assays verified the interactions between VP088 and VP018, VP068, or VP156 *in vitro*. Moreover, confocal microscopy analysis showed that VP088 markedly altered the cellular distribution of exogenously expressed VP018 and VP068 and ultimately translocated into virus assembly sites together upon SGIV infection. Differently, VP088 mostly co-localized with exogenous VP156 in co-transfected cells and almost simultaneously translocated into the virus assembly sites, suggesting that VP088 participated in SGIV replication through interactions with other viral proteins in different ways. Interestingly, VP088 also abrogated IFN response induced by grouper (*Epinephelus coioides*) EccGAS-EcSTING and EcTBK1 *in vitro*. Co-IP assays showed that VP088 interacted with EccGAS, EcSTING, EcTBK1, or EcIRF3, while only degraded EcTBK1 via Ecp62-mediated autophagic degradation. Furthermore, VP088 decreased EcTBK1-induced EcIRF3 phosphorylation and nuclear translocation. In addition, the ectopic expression of VP088 attenuated the antiviral function of EcSTING/EcTBK1/EcIRF3 against red-spotted grouper nervous necrosis virus (RGNNV) infection. Thus, our results not only identified the association between SGIV VP088 and other viral proteins during replication, but also for the first time demonstrated that an iridoviral envelope protein could function as an immune evasion protein via abrogating EcTBK1-induced interferon response.

**IMPORTANCE:**

Iridovirus infection frequently causes high levels of morbidity and mortality among commercially and ecologically important fish, crustaceans, amphibians, and reptiles. However, the molecular mechanism of iridovirus pathogenesis still remains largely unknown, and few effective countermeasures have been developed to date. Using the Singapore grouper iridovirus (SGIV) infection model *in vitro*, we identified the potential viral proteins that interacted with envelope protein VP088 during virus replication. Moreover, for the first time, we demonstrated that VP088 interacted with EccGAS, EcSTING, EcTBK1, and EcIRF3, but only degraded EcTBK1 via Ecp62-mediated autophagic degradation, thereby inhibiting the host IFN response. Thus, our results not only contribute to elucidating the mechanism of SGIV pathogenesis but also provide a novel molecular target for the construction of immunogenic live vaccines against iridoviral diseases in the future.

## INTRODUCTION

Innate immunity is an important first-line defense against various pathogens. Upon virus invasion, host cell pattern recognition receptors (PRRs) recognize pathogen-associated molecular patterns of these viruses and trigger a cascade of signal transduction to activate interferon-mediated innate immune responses (1–3). Among these PRRs, cyclic GMP-AMP synthase (cGAS) senses cytoplasmic double-stranded DNA, stimulating its own activity to form cGAMP (4), and then cGAMP acts as a second messenger to activate the adaptor stimulator of interferon genes (STING) (5). Subsequently, STING recruits TANK-binding kinase 1 (TBK1) and IFN regulatory factor 3 (IRF3), leading to their phosphorylation (6). The phosphorylated IRF3 forms dimers and translocates into the nucleus and finally induces the expression of the interferon-stimulated genes (ISGs) to resist the invading pathogens, especially DNA viruses (7).

During the co-evolution between virus and host, viruses have also developed various strategies to evade host immune attack for efficient replication (8, 9). For DNA viruses, the increasing amount of evidence reveals that multiple viral proteins are able to abrogate the host IFN immune response by interfering with the normal functions of key molecules in the cGAS-STING signaling pathway (10–12). For instance, herpes simplex virus 1 (HSV-1) VP22 inhibited the enzymatic activity of cGAS (13), while UL41 targeted the degradation of cGAS to evade the immune response mediated by cGAS-STING (14). HSV-1 UL38 also inhibited the binding of cGAMP to STING through direct interaction with STING, thereby blocking the formation of STING-TBK1-IRF3 complex and antagonizing the IFN immune response (15). Notably, structurally related herpesvirus tegument proteins, such as VP22 and UL37, not only played roles in viral entry and viral capsid transportation but also suppressed the type I IFN signaling pathway and other innate immune responses (13, 16). Moreover, the deletion of such viral immune evasion genes might provide a novel strategy for constructing safe and immunogenic live vaccines against infectious pathogens (17).

Singapore grouper iridovirus (SGIV), a novel member of the genus *Ranavirus*, is a highly pathogenic DNA virus isolated from the spotted-grouper (*Epinephelus tauvina*), causing more than 90% mortality in juvenile grouper (18–20). The high pathogenicity might be partially due to its ability to disrupt the host antiviral immune response (21). Consistently, recent studies have demonstrated that several viral proteins from SGIV abrogate host antiviral immune response for efficient replication (22–25). For instance, VP131 interacted with EcSTING and degraded it through both the autophagy-lysosome pathway and the ubiquitin-proteasome pathway (24). Similarly, VP018 degraded EcSTING, EcTBK1, and EcIRF3 proteins *in vitro* and reduced their induction of interferon response (25). Similar to HSV-1, SGIV also encoded multiple structural proteins. Among them, VP088 was identified as an envelope protein involved in virus entry (26), and deletion of VP088 significantly reduced the infectivity of SGIV, but it showed no obvious effects on genomic stability, replication, and release of progeny viruses during SGIV infection (26, 27). Whether VP088 exerted other functions during SGIV-host interactions remained uncertain.

In this study, we first screened the potential viral proteins that interacted with VP088 during SGIV infection using GFP pull-down and MS analysis and verified their interactions using co-immunoprecipitation (Co-IP) assay. Based on the results that VP088 interacted with VP018 and VP018 abrogated host interferon response during SGIV infection (25), we also clarified the regulatory roles of VP088 on EcSTING-EcTBK1-mediated antiviral IFN-I signaling. Our results will not only shed new light on understanding the roles of structural proteins in fish virus infection but also provide novel molecular targets for the development of vaccines against iridoviral diseases in the future.

## MATERIALS AND METHODS

### Cells and viruses

Grouper spleen (GS) cells were established by our laboratory and subcultured in Leibovitz’s L15 medium (Gibco) supplemented with 10% fetal bovine serum (Excell) at 28°C (28). SGIV and red-spotted grouper nervous necrosis virus (RGNNV) were propagated in GS cells. Virus stocks were stored at −80°C until used.

### Antibodies and reagents

The anti-HA and anti-Flag antibodies were purchased from Cell Signaling. Anti-GFP and anti-β-tubulin antibodies were purchased from Abcam. Rabbit against SGIV VP018, VP068, and VP156 polyclonal antibodies and mouse against VP088 and RGNNV capsid protein (CP) antibodies were prepared by Wuhan GeneCreate Biological Engineering Co., Ltd (China) (21, 28). The anti-IRF3, anti-p-IRF3, and anti-Lamin B1 antibodies were purchased from ABclonal. The anti-p62 antibody was obtained from Selleck. HRP-conjugated anti-HA and anti-Flag were purchased from AlpalifeBio. Peroxidase-conjugated goat anti-mouse or anti-rabbit IgG, Alexa Fluor 555 goat anti-mouse IgG (H+L), and Alexa Fluor 488 goat anti-rabbit IgG (H+L) were purchased from Thermo Fisher Scientific.

MG132 (ubiquitin-proteasome inhibitor), NH_4_Cl (lysosome inhibitor), and 4,6-diamidino-2-phenylindole (DAPI) were purchased from Sigma Aldrich, and 3-MA (autophagy inhibitor) was purchased from Selleck. Paraformaldehyde (PFA) was purchased from Thermo Fisher Scientific. The stock of NH_4_Cl and 3-MA was prepared using sterile water, and MG132 was diluted with dimethyl sulfoxide.

### Plasmid construction

The full-length SGIV VP088 (GenBank accession no. YP_164183.1) was amplified by PCR from SGIV genomic DNA. To investigate the subcellular localization and function of VP088, the full-length ORF of VP088 was subcloned into pcDNA3.1-HA-N (Clontech), pCMV-3×Flag-N (Clontech), pEGFP-C1 (Clontech), and pmCherry-C1 (Clontech), respectively. The recombinant plasmids, including pcDNA3.1-HA-VP088 (pHA-VP088), pCMV-3×Flag-VP088 (pFlag-VP088), pEGFP-C1-VP088 (pGFP-VP088), and pmCherry-C1-VP088 (pmCherry-VP088), were validated by sequencing. The full-length ORF of Ecp62 was subcloned into pcDNA3.1-HA-N (Clontech) to obtain pcDNA3.1-HA-Ecp62 (pHA-Ecp62). The corresponding primers used in this study were listed in Table S1 (Table S1).

The recombinant plasmids, including pHA-VP018, pHA-VP068, pHA-VP156, pHA-EcSTING, pHA-EcTBK1, pHA-EcTBK1-∆C, pHA-EcTBK1-∆N, pHA-EcIRF3, pFlag-EccGAS, pFlag-EcSTING, pFlag-EcTBK1, pGFP-VP018, pGFP-VP068, pGFP-VP156, pGFP-EccGAS, pGFP-EcSTING, pGFP-EcTBK1, and pGFP-EcIRF3, were described previously (23, 24, 29).

### Cell transfection and virus infection

GS cells were seeded in 24-well plates or dishes overnight and transfected with the indicated plasmids using Lipofectamine 2000 (Thermo Fisher Scientific) according to the manufacturer’s instructions. To screen the potential viral or cellular proteins that interacted with VP088, GS cells were seeded into 10 cm² dishes and then transfected with pGFP-C1 or pGFP-VP088. At 24 h post-transfection, cells were infected with SGIV and then collected for Co-IP assays and LC-MS/MS analysis (Guangzhou Fitgene Biotechnology Co., Ltd., China) as described previously (30).

To evaluate the effect of VP088 on the expression of IFN-related genes induced by EccGAS/EcSTING or EcTBK1, GS cells were co-transfected with pHA-VP088 and pFlag-EccGAS plus pGFP-EcSTING or pGFP-EcTBK1, respectively. At 48 h post-transfection, cells were collected for quantitative PCR (qPCR) analysis.

In addition, to clarify whether VP088 affected the antiviral activities of EcSTING, EcTBK1, or EcIRF3, GS cells were co-transfected with pHA-EcSTING, pHA-EcTBK1, or pHA-EcIRF3, together with either pFlag-VP088 or the empty vector pCMV-3×Flag. At 24 h post-transfection, cells were infected with RGNNV (which lacks VP088 or its homolog). The infected cells were harvested for subsequent qPCR and immunoblotting (IB) analysis.

### Subcellular localization

To observe the localization of VP088 and its interacting viral proteins, GS cells were seeded into glass bottom cell culture dishes (35 mm) and co-transfected with 0.5 µg pmCherry-VP088 and 0.5 µg pGFP-VP018, pGFP-VP068, or pGFP-VP156, respectively. To examine the trafficking events of these viral proteins during SGIV infection, the co-transfected cells were infected with SGIV at 24 h post-transfection and then harvested after another 24 h incubation.

To detect the localization of VP088 and its interacting cellular proteins, GS cells were co-transfected with pmCherry-VP088 and pGFP-EccGAS, pGFP-EcSTING, pGFP-EcTBK1, or pGFP-EcIRF3, respectively. At 48 h post-transfection, cells were fixed and stained with DAPI for 10 min. All samples were imaged under a confocal laser scanning microscope (CLSM, Leica) equipped with an oil-immersion objective lens (63×).

### Indirect immunofluorescence assay (IFA)

To determine the intracellular distribution of viral proteins during infection, IFA was carried out using specific antibodies against different viral proteins as described previously (24). In brief, GS cells were seeded into 35 mm glass bottom cell culture dishes overnight and infected with SGIV for 12 or 24 h. Then, the cells were fixed by 4% PFA for 1 h and then permeabilized with 0.1% Triton X-100 for 15 min at room temperature. After blocking with 2% bovine serum albumin, cells were incubated with primary antibodies for 2 h. The primary antibodies used included anti-HA (1:3,000), anti-VP088 (1:1,000), anti-VP018 (1:1,000), anti-VP068 (1:1,000), and anti-VP156 (1:1,000). Then, cells were washed and incubated with Alexa Fluor 555-conjugated goat anti-mouse IgG (1:800) or Alexa Fluor 488-conjugated goat anti-rabbit IgG (1:800) for another 2 h. Finally, the nuclei were stained with DAPI, and the samples were observed under a CLSM.

### Dual-luciferase reporter assay

To evaluate the effect of SGIV VP088 on cellular IFN immune response, the IFN1, IFN3, and ISRE promoter activities were determined using dual-luciferase reporter assay as described previously (24). Briefly, GS cells were seeded into 12-well plates overnight and then co-transfected with pHA-VP088 (or the empty vector control), along with pGFP-EcTBK1 (or the combination of pFlag-EccGAS and pGFP-EcSTING), reporter gene plasmid (pIFN1-Luc, pIFN3-Luc, or pISRE-Luc), and internal control plasmid pRL-SV40 (Promega). At 24 h post-transfection, cells were harvested and lysed to detect the luciferase activities using a Dual-Luciferase Reporter System (Promega) according to the manufacturer’s manual. Luciferase activities were read using a microplate reader from Tecan (Switzerland).

### RNA interference

To evaluate the effect of VP088 knockdown on IFN immune response during SGIV infection, three specific small interfering RNA (siRNA) oligonucleotides targeting VP088 (si-VP088) and stealth RNAi negative control (siRNA-NC) were designed by Sangon Biotech (Shanghai), and the specific sequences were listed in Table S1. GS cells were transfected with si-VP088-1, si-VP088-2, si-VP088-3, or siRNA-NC for 24 h and then infected with SGIV for another 24 h. The silencing efficiency of si-VP088 was determined by western blotting using anti-VP088 (1:1,000), and si-VP088-1 was screened as the optimal candidate. Subsequently, GS cells transfected with si-VP088-1 (160 nM/well) or siRNA-NC were infected with SGIV and harvested at 24 h post-infection (p.i.) for qPCR analysis.

To determine whether Ecp62 was involved in VP088-mediated degradation of EcTBK1, three specific si-RNAs targeting Ecp62 (si-Ecp62) were synthesized by Sangon Biotech (Shanghai), and the silencing efficiency was determined by IB analysis. Subsequently, GS cells were transfected with si-Ecp62-1, pHA-EcTBK1, and pFlag-VP088 for 48 h and then harvested for IB analysis.

### RNA isolation and qPCR

Total RNA was extracted from cells using the Cell Total RNA Isolation Kit (FORE GENE) according to the manufacturer’s instructions and then reverse transcribed using the ReverTra Ace qPCR RT Kit (TOYOBO). Subsequently, a qPCR assay was performed using the 2× PolarSignal SYBR Green qPCR Mix (MIKX) under an Applied Biosystems QuantStudio 5 Real Time Detection System (Thermo Fisher Scientific). The qPCR conditions were as follows: 94°C for 20 s, followed by 40 cycles at 94°C for 10 s, 55°C for 10 s, and 72°C for 10 s. The relative expression levels (fold change) were calculated with the 2^−ΔΔCt^ method using *β-actin* as an internal control. The IFN-related genes, including ISG15, ISG56, Viperin, and myxovirus resistance gene (MX1), were detected, and the corresponding primers were used as described previously (24).

### Co-IP assay

The interactions between VP088 and key molecules (including VP018, VP068, VP156, EccGAS, EcSTING, EcTBK1, EcTBK1-∆C, EcTBK1-∆N, EcIRF3, and Ecp62) were carried out using Co-IP assay as described previously (24). Briefly, GS cells were seeded into 10 cm^2^ dishes overnight and then co-transfected with pHA-VP088, pFlag-VP088, or pGFP-VP088 and pHA-VP018, pHA-VP068, pHA-VP156, pFlag-EccGAS, pHA-EcSTING, pHA-EcTBK1, pHA-EcTBK1-∆C, pHA-EcTBK1-∆N, pHA-EcIRF3, pHA-Ecp62, or empty vectors, respectively. At 48 h post-transfection, cells were collected and lysed by IP lysis buffer (Thermo Fisher Scientific). The whole cell lysis buffer (WCLs) was centrifuged at 12,000 × *g* for 5 min, and the supernatants were collected for immunoprecipitation using the HA-tag Protein IP Assay Kit with Magnetic Beads (Beyotime), Flag-tag Protein IP Assay Kit with Magnetic Beads (Beyotime), and ChromoTek GFP-Trap Magnetic Particles Kit (Proteintech). These immunoprecipitated proteins and WCLs were further subjected to IB analysis using antibodies as indicated.

### IB analysis

For IB analysis, the immunoprecipitated proteins and WCLs were separated by 10% SDS-PAGE and then transferred onto 0.2 µm polyvinylidene difluoride membranes (Millipore). The membranes were blocked with 5% skim milk in tris-buffer saline with 0.5% Tween-20 (TBST) for 2 h at room temperature. Subsequently, the membranes were incubated with the indicated primary antibodies, including anti-GFP (1:5,000), anti-HA (1:5,000), anti-Flag (1:5,000), anti-VP088 (1:1,000), anti-p62 (1:1,000), anti-IRF3 (1:1,000), anti-p-IRF3 (1:1,000), anti-Lamin B1 (1:1,000), anti-HA (HRP) (1:1,000), anti-Flag (HRP) (1:1,000), anti-RGNNV CP (1:2,000), or anti-β-tubulin (1:5,000) for another 2 h. After washing, the membranes were incubated with secondary antibodies, such as horseradish peroxidase-conjugated goat anti-mouse or anti-rabbit IgG (1:10,000). Finally, the membranes were visualized with the ECL chemiluminescence solution, and the intensities of protein bands were quantified using Image J software. Data were representative of three independent experiments.

### Nucleocytoplasmic separation

To clarify whether VP088 affected the nuclear translocation of EcIRF3, GS cells were transfected with pFlag-EcTBK1 alone or co-transfected with pGFP-VP088 for 48 h. The nucleocytoplasmic separation was performed using the Nuclear and Cytoplasmic Protein Extraction Kit (Beyotime) as described previously (25), and then the different fractions were subjected to IB assay.

### Statistical analyses

All statistical analyses were carried out using GraphPad Prism Software. The obtained data were expressed as mean ± standard deviation. Differences between two groups were calculated using two-tailed Student t-tests. *P* < 0.05 was considered statistically significant (*P < 0.05).

## RESULTS

### VP088 interacted with VP018/VP068/VP156

**Fig 1:**
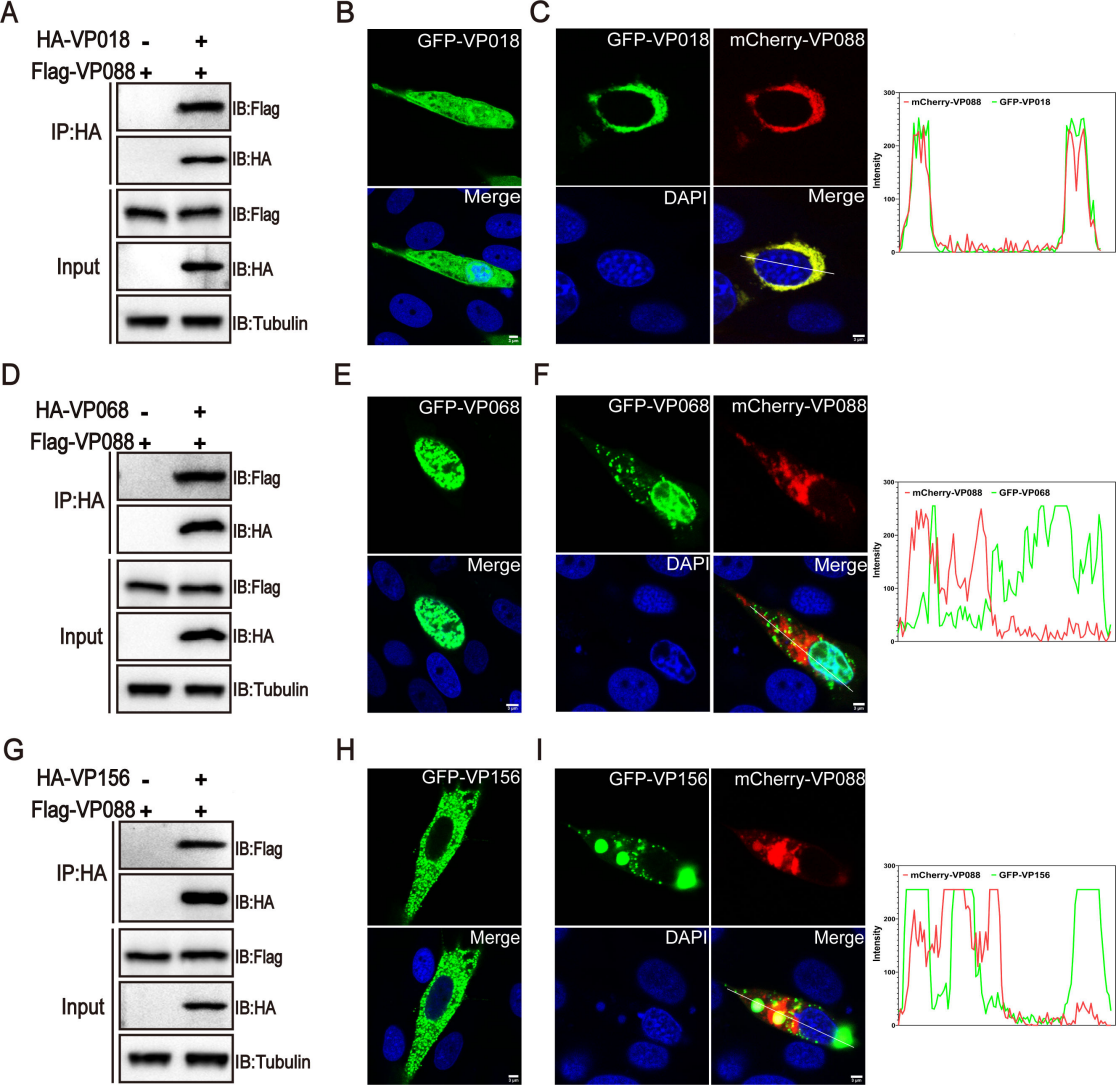
Identification of potential viral proteins interacted with VP088. (**A, D, G**) The interactions between VP088 and VP018 (**A**), VP068 (**D**), or VP156 (**G**). GS cells were co-transfected with pFlag-VP088 and pHA-VP018, pHA-VP068, or pHA-VP156, respectively. At 48 h post-transfection, cells were collected for Co-IP assays and western blotting analysis. (**B, E, H**) Subcellular localization of VP018 (**B**), VP068 (**E**), or VP156 (**H**) alone in transfected cells. (**C, F, I**) Co-localization analysis of VP018 (**C**), VP068 (**F**), and VP156 (**I**) and VP088 *in vitro*. GS cells were co-transfected with pmCherry-VP088 and pGFP-VP018, pGFP-VP068, or pGFP-VP156 and then stained with DAPI at 48 h post-transfection for visualization under a confocal microscope. Scale bar, 3 µM.

As an envelope protein, SGIV VP088 was demonstrated to be involved in viral assembly and function as an anchor protein during SGIV infection (31). Thus, we identified the potential proteins that interact with VP088 using GFP pull-down assay, followed by MS analysis (Table S2). The MS results showed that VP088 was associated with viral proteins including SGIV VP018, VP068, and VP156. Here, Co-IP assays verified the interactions between VP088 and VP018, VP068, or VP156 *in vitro* without SGIV infection (Fig. 1A, D, and G). Moreover, confocal microscopy analysis showed that VP088 altered the intracellular distribution of VP018 and VP068 in co-transfected cells. In detail, the fluorescence of VP018 alone was present throughout the cells in both the nucleus and the cytoplasm. However, the co-expression of VP088 prevented the trafficking of VP018 from the cytoplasm into the nucleus (Fig. 1B and C). Differently, VP088 co-expression only partially prevented the transport of the fluorescence of VP068 from the cytoplasm into the nucleus, and a small fraction of fluorescence in the cytoplasm was overlapped between VP088 and VP068 (Fig. 1E and F). In addition, the fluorescence of VP156 was localized in the cytoplasm and partially overlapped with that of VP088 (Fig. 1H and I).

### VP088 and its interacting proteins were involved in virus assembly

**Fig 2:**
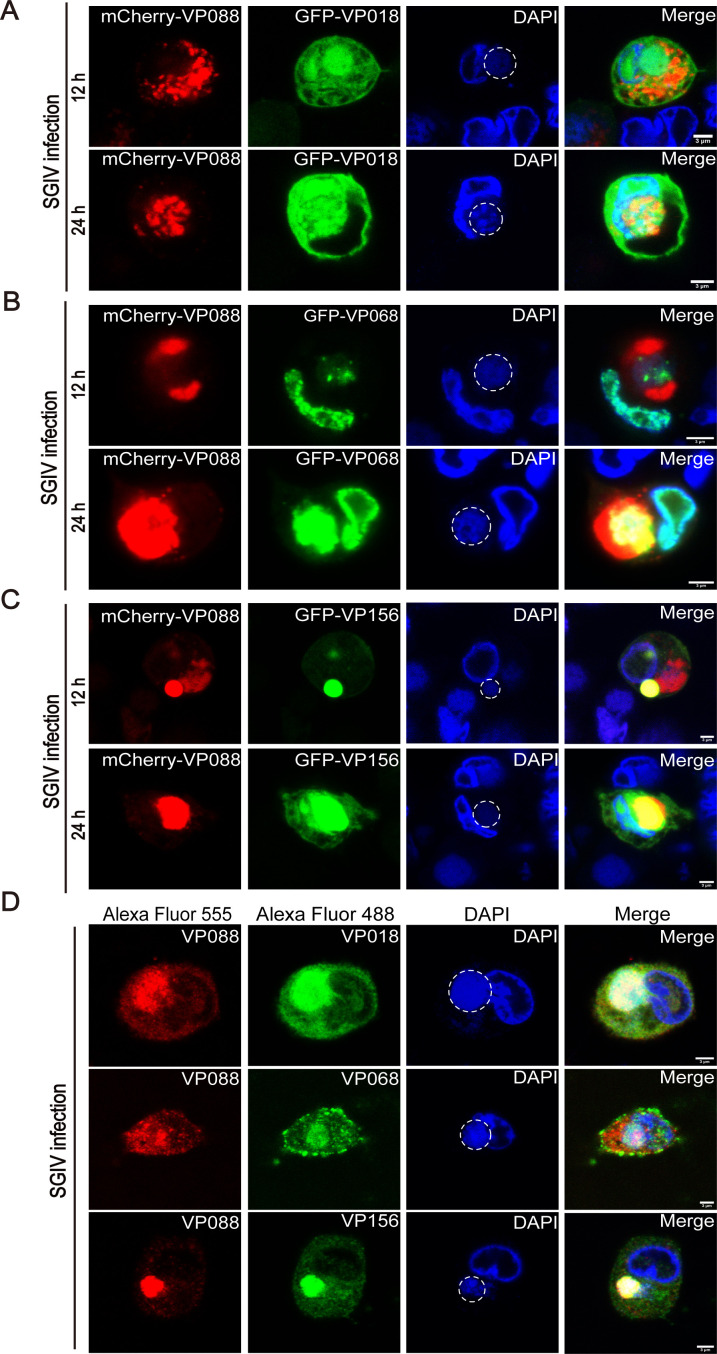
Involvement of VP088 and its interacting viral proteins in virus assembly. (**A–C**) Co-localization analysis of VP088 and VP018 (**A**), VP068 (**B**), or VP156 (**C**) in GS cells during SGIV infection. GS cells were co-transfected with pmCherry-VP088 and pGFP-VP018, pGFP-VP068, or pGFP-VP156, respectively. At 24 h post-transfection, cells were infected with SGIV for 12 or 24 h. Then, cells were fixed and stained with DAPI for visualization under a confocal microscope. DAPI staining (blue) showed the extranuclear viral DNA which indicated the virus assembly sites and cellular DNA. The circles indicate the virus assembly sites. (**D**) Intracellular distribution of endogenous VP088 and VP018, VP068, or VP156 during SGIV infection. GS cells were infected with SGIV for 24 h and then collected for IFA using specific antibodies against VP088, VP018, VP068, and VP156. The samples were visualized under a confocal microscope. The circles indicate the virus assembly sites. Scale bar, 3 µM.

To clarify whether VP088-interacting viral proteins were involved in virus assembly, GS cells were co-transfected with pmCherry-VP088 and pGFP-VP018, pGFP-VP068, or pGFP-VP156 and then incubated with SGIV for 12 or 24 h, respectively. Extranuclear DAPI staining was used to identify the sites of viral DNA replication indicative of virus assembly sites (32). As shown in Fig. 2, VP018 and VP068 were partially translocated into the virus assembly sites before VP088 at 12 h p.i., although both of them were present there at 24 h p.i. (Fig. 2A and B). Differently, the fluorescence of VP156 and VP088 was almost simultaneously present in the virus assembly sites, and their abundances were both increased at 24 h p.i. (Fig. 2C). Using the specific antibodies, we found that the majority of fluorescence from endogenous VP018, VP068, VP156, and VP088 proteins was all present in the virus assembly sites at 24 h p.i. The minority of fluorescence was labeled on the viral particles which were present throughout the cytoplasm (Fig. 2D). Thus, our results showed that VP088 might participate in SGIV replication through the interactions with other viral proteins in different ways.

### VP088 abrogated the host IFN response

**Fig 3:**
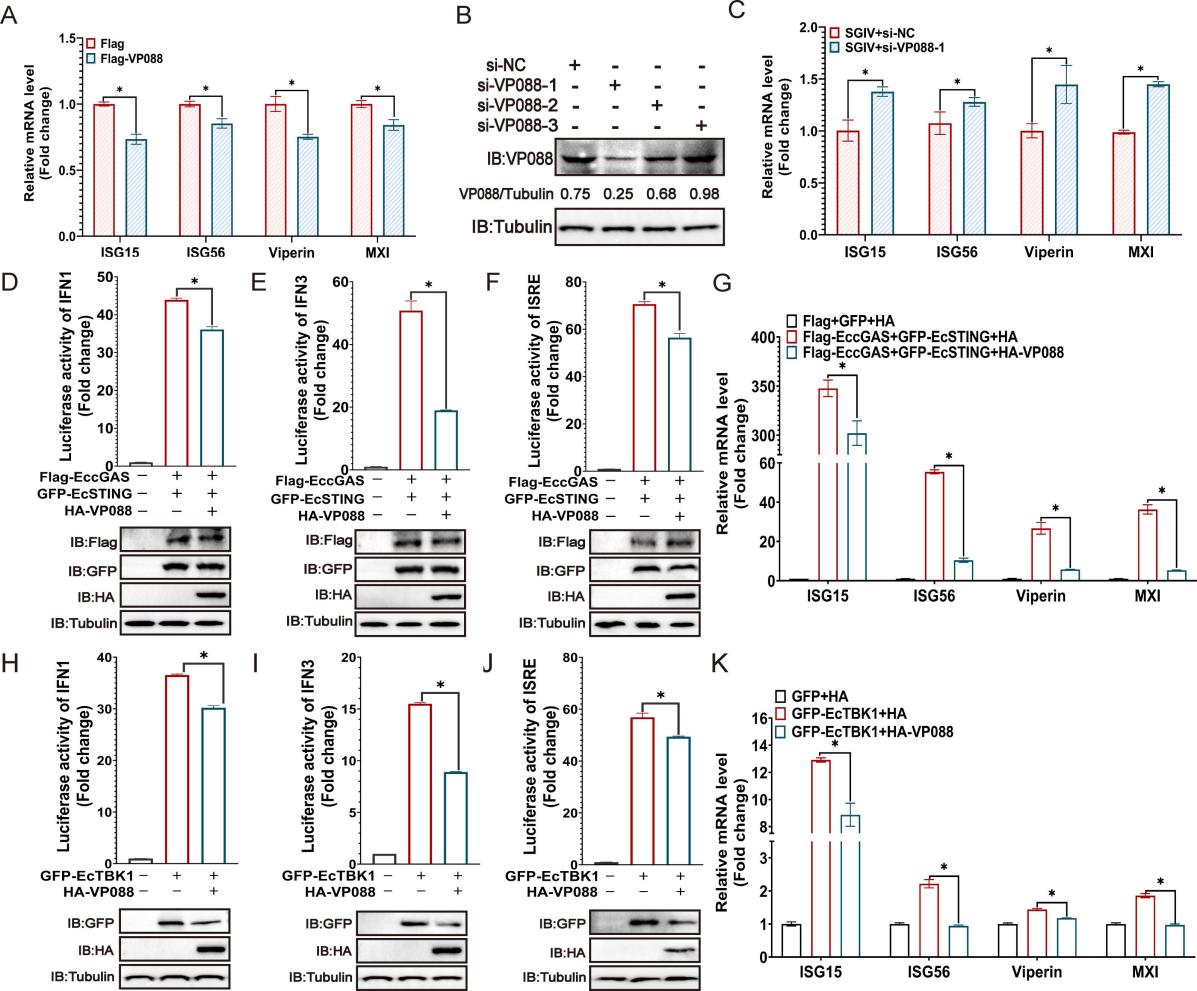
VP088 down-regulated host IFN response. (**A**) VP088 overexpression alone decreased the transcription levels of IFN- related genes. The mRNA levels of ISG15, ISG56, Viperin, and MX1 in pFlag-VP088-transfected GS cells were measured using qPCR. (**B**) The silencing effects of siRNA on VP088 protein synthesis during SGIV infection. GS cells transfected with siRNA-NC, si-VP088-1, si-VP088-2, or si-VP088-3 were infected with SGIV for 24 h and then collected for IB analysis using anti-VP088 antibody. (**C**) The transcription levels of IFN-related genes in VP088 silenced cells upon SGIV infection. GS cells were transfected with si-VP088 and then infected with SGIV for 24 h. Cells were collected for qPCR analysis to evaluate the mRNA levels of ISG15, ISG56, Viperin, and MX1. (**D–F**) VP088 decreased EccGAS-EcSTING-induced promoter activities of IFN1 (**D**), IFN3 (**E**), or ISRE (**F**). (**H–J**) VP088 reduced EcTBK1-induced promoter activities of IFN1 (**H**), IFN3 (**I**), or ISRE (**J**). Cells were co-transfected with the reporter gene plasmids (IFN1-Luc, IFN3-Luc, or ISRE-Luc), internal control plasmid pRL-SV40, pHA-VP088 and pFlag-EccGAS plus pGFP-EcSTING, or pGFP-EcTBK1 for 24 h, and then collected for dual fluorescence reporter gene assay. (**G and K**) VP088 decreased EccGAS-EcSTING (**G**) and EcTBK1 (**K**) induced IFN response. Cells were transfected with pHA-VP088 and pFlag-EccGAS plus pGFP-EcSTING or pGFP-EcTBK1 for 48 h, and then the mRNA levels of IFN-related genes were detected using qPCR. *P < 0.05.

Given that VP088 interacted and altered the localization of VP018 without virus infection, and VP018 abrogated the host IFN response for efficient replication (25), we speculated that VP088 might also antagonize host innate immune defenses during virus infection. Interestingly, our findings showed that VP088 overexpression alone down-regulated the host IFN response (Fig. 3A). To assess the effect of VP088 knockdown on host response, we designated the specific siRNAs for VP088 and first detected their silencing effects on VP088 protein synthesis during SGIV infection. According to the knockdown efficacy, si-VP088-1 was chosen for the following experiment (Fig. 3B). Consistently, transfection of si-VP088-1 led to the restoration of the IFN response compared with wild-type SGIV-infected cells (Fig. 3C). Moreover, VP088 overexpression not only significantly down-regulated the promoter activities of IFN1 (Fig. 3D and H), IFN3 (Fig. 3E and I), and ISRE (Fig. 3F and J) activated by EccGAS+EcSTING and EcTBK1 but also markedly decreased the transcription levels of ISG15, ISG56, Viperin, and MX1 induced by these proteins (Fig. 3G and K). Collectively, SGIV VP088 was speculated to abrogate EccGAS-EcSTING-EcTBK1-activated host IFN response *in vitro*.

### VP088 targeted EcTBK1 to regulate the IFN signaling

**Fig 4:**
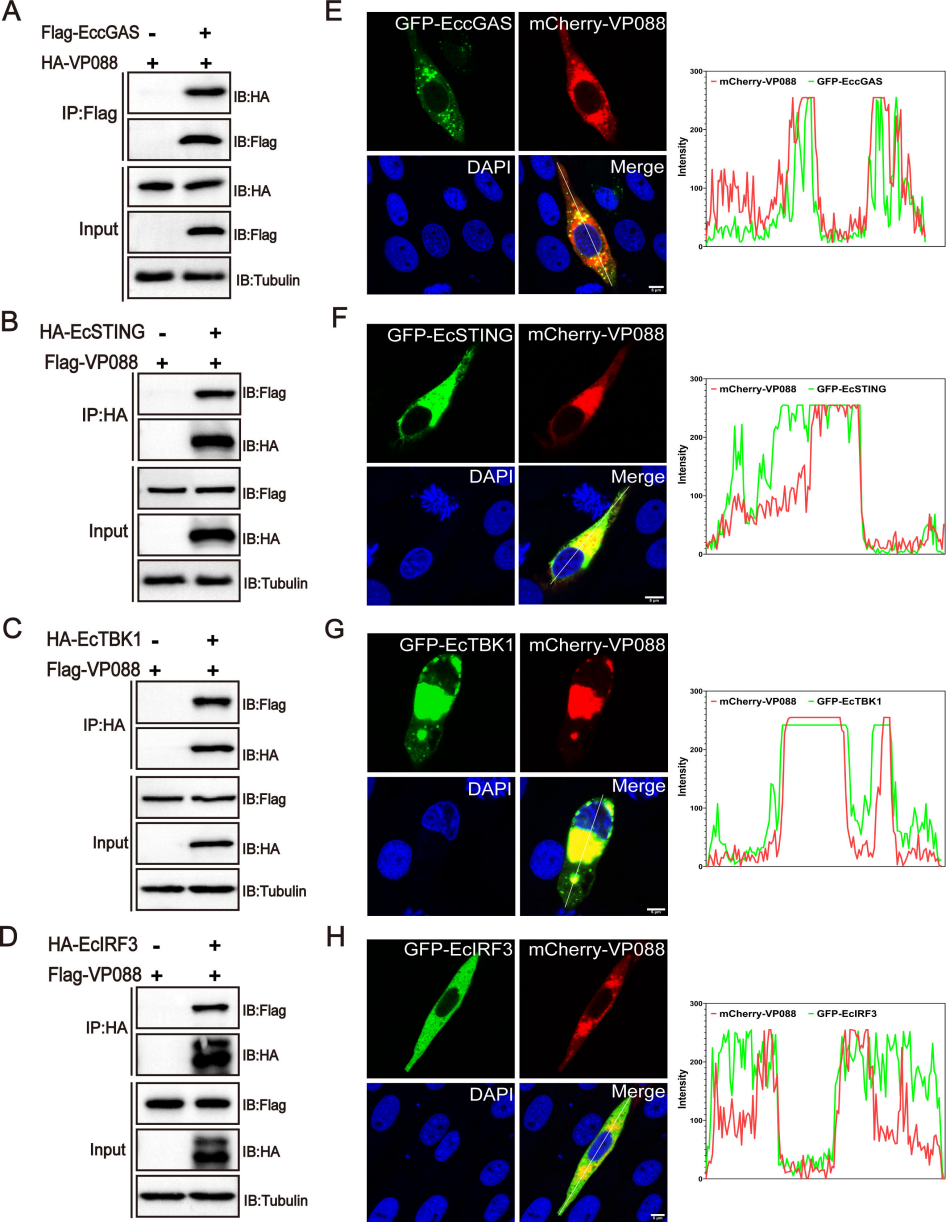
Identification of VP088-interacting proteins in the EcSTING-EcTBK1 signaling pathway. (**A–D**) The interactions between VP088 and EccGAS (**A**), EcSTING (**B**), EcTBK1 (**C**), or EcIRF3 (**D**) *in vitro*. GS cells were co-transfected with VP088 (or empty vector) and EccGAS, EcSTING, EcTBK1, or EcIRF3 for 48 h and then collected for Co-IP assay and western blotting analysis. (**E–H**) Co-localization analysis of VP088 and EccGAS (**E**), EcSTING (**F**), EcTBK1 (**G**), or EcIRF3 (**H**) in co-transfected cells. GS cells were co-transfected with VP088 and EccGAS, EcSTING, EcTBK1, or EcIRF3 and then stained with DAPI at 48 h post-transfection visualization under a confocal microscope. Scale bar, 5 µM.

To clarify the critical molecules hijacked by VP088 to regulate the IFN response, we first performed Co-IP assay and western blotting analysis *in vitro*. GS cells were co-transfected with VP088 and EccGAS, EcSTING, EcTBK1, or EcIRF3, respectively. As shown in Fig. 4A, Flag-tagged EccGAS interacted with HA-tagged VP088 but not HA peptide when they were co-expressed in GS cells. Similarly, HA-tagged EcSTING (Fig. 4B), EcTBK1 (Fig. 4C), and EcIRF3 (Fig. 4D) all interacted with Flag-tagged VP088 but not Flag peptide when they were co-transfected in GS cells. Further, confocal microscopic observation revealed that the fluorescence signal from VP088 almost overlapped with those from EccGAS, EcSTING, EcTBK1, or EcIRF3 in co-transfected cells (Fig. 4E through H). Thus, VP088 was elucidated to interact with EccGAS, EcSTING, EcTBK1, and EcIRF3 *in vitro*.

**Fig 5:**
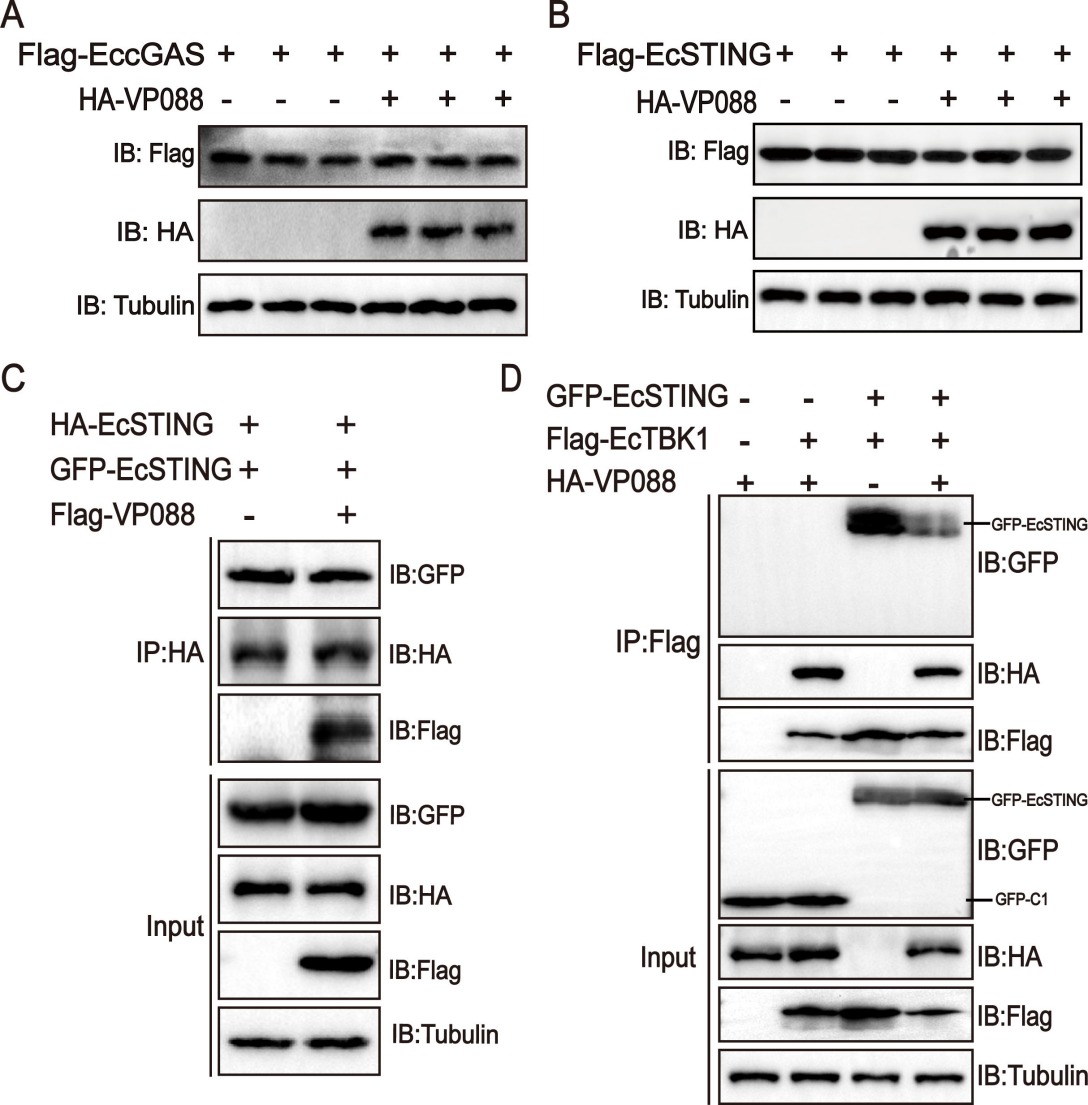
VP088 targeted EcTBK1 to regulate the IFN response. (**A and B**) The effect of VP088 on the stability of exogenous EccGAS (**A**) and EcSTING (**B**) in transfected cells. GS cells were co-transfected with pHA-VP088 and pFlag-EccGAS or pFlag-EcSTING and then collected for western blotting analysis. (**C**) The effect of VP088 on STING dimerization. GS cells were transfected with pFlag-VP088, pHA-EcSTING, and pGFP-EcSTING, and then, cells were collected at 24 h post-transfection for Co-IP assay and western blotting analysis. (**D**) The effect of VP088 on the assembly of EcSTING-EcTBK1 complex. GS cells were transfected with pHA-VP088, pGFP-EcSTING, and pFlag-EcTBK1 and then collected for Co-IP assay and western blotting analysis.

Next, we examined the potential effect of VP088 on EccGAS-EcSTING in co-transfected cells. As shown in Fig. 5, VP088 overexpression showed no obvious effect on the stability of the exogenous EccGAS and EcSTING in transfected cells (Fig. 5A and B). Moreover, VP088 also did not affect the dimerization of EcSTING (Fig. 5C). Notably, once EcSTING and EcTBK1 were co-expressed, the formation of EcSTING-EcTBK1 complex was markedly reduced after VP088 overexpression (Fig. 5D). Thus, it was proposed that VP088 might target EcTBK1 but not EccGAS/EcSTING to regulate the IFN signaling.

### Ecp62 was essential for VP088-mediated autophagic degradation of EcTBK1

**Fig 6:**
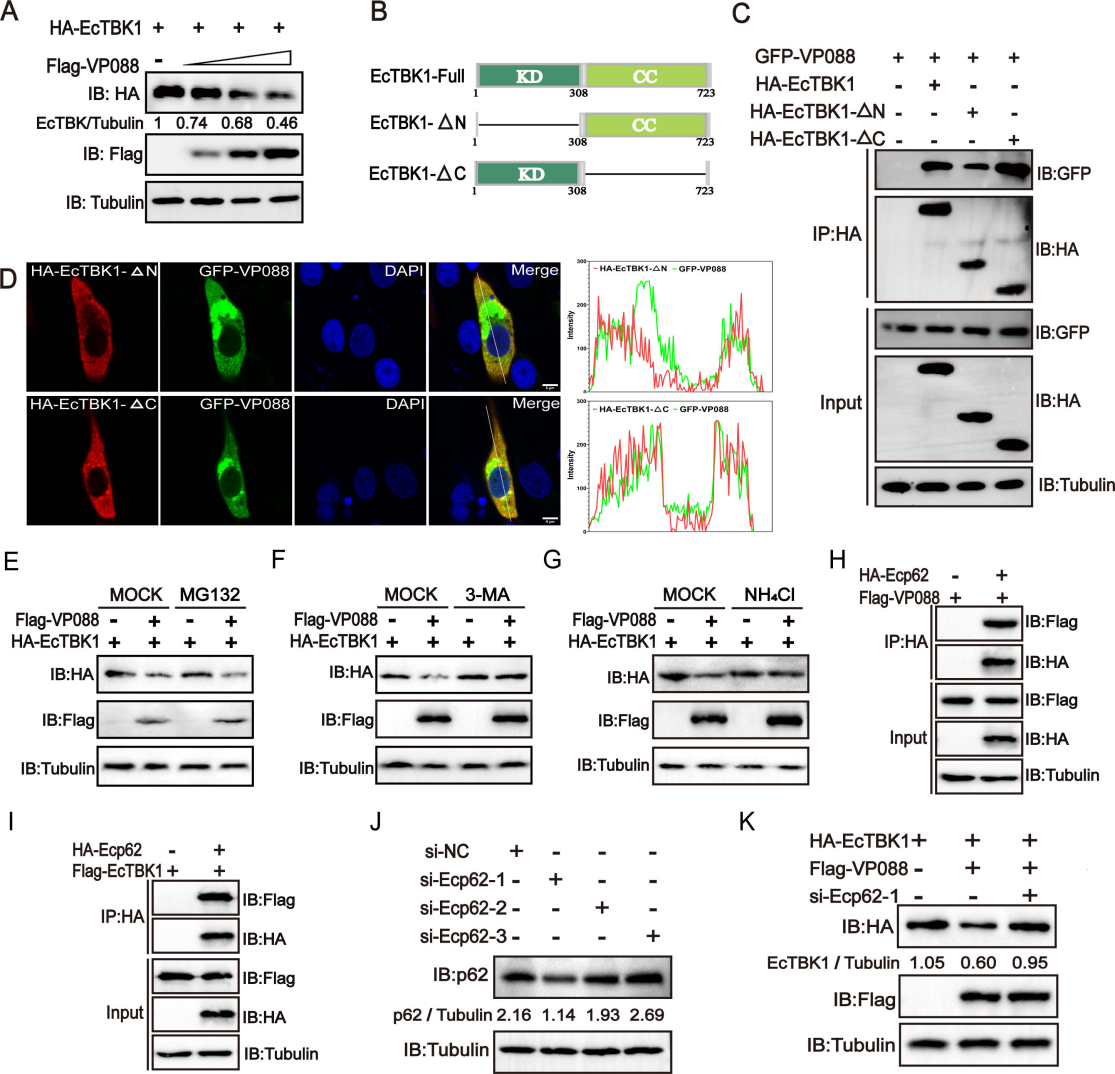
Involvement of Ecp62 in VP088-mediated autophagic degradation of EcTBK1. (**A**) VP088 degraded EcTBK1 in a dose-dependent manner. GS cells were co-transfected with pHA-EcTBK1 and pFlag-VP088 under different concentrations, and then the cells were collected for western blotting analysis. (**B**) The schematic diagram of the truncated mutants of EcTBK1. (**C**) The interaction between VP088 and different domains of EcTBK1. Cells were transfected with pGFP-VP088 and pHA-EcTBK, pHA-EcTBK1-∆C, or pHA-EcTBK1-∆N, and then the cells were collected for Co-IP assay and western blotting analysis. (**D**) Co-localization analysis between VP088 and different truncations of EcTBK1 in GS cells. Cells were transfected with pGFP-VP088 and pHA-EcTBK1-∆C or pHA-EcTBK1-∆N and then fixed at 48 h post-transfection for IFA. The samples were visualized under a confocal microscope. Scale bar, 5 µM. (**E–G**) The effects of different inhibitors, including MG132 (**E**), 3-MA (**F**), or NH_4_Cl (**G**), on the degradation effect of VP088 on exogenous EcTBK1. GS cells were co-transfected with pFlag-VP088 and pHA-EcTBK1, then treated without or with MG132, 3-MA, or NH_4_Cl, and collected for western blotting. (**H, I**) The interactions between Ecp62 and VP088 (**H**) or EcTBK1 (**I**) *in vitro*. GS cells were transfected with Ecp62 and VP088 or EcTBK1 and then collected for Co-IP assay and western blotting assay. (**J**) The silencing effect of siRNA on Ecp62 protein synthesis. GS cells were transfected with siRNA-NC, si-Ecp62-1, si-Ecp62-2, or si-Ecp62-3 and then collected for western blotting analysis. (**K**) Ecp62 was essential for the degradation effect of VP088 on EcTBK1. GS cells were co-transfected with si-Ecp62-1, pFlag-VP088, and pHA-EcTBK1, and then the cells were collected for western blotting analysis.

Overexpression of VP088 markedly decreased the abundance of EcTBK1 in co-transfected cells in a dose-dependent manner (Fig. 6A). To identify the key domains of EcTBK1 involved in its interaction with VP088, we constructed two EcTBK1 truncating mutants, including EcTBK1-∆C (lacking the C-terminal, amino acids 1–308) and EcTBK1-∆N (lacking the N-terminal transmembrane region, amino acids 309–723) (Fig. 6B). Interestingly, VP088 was immunoprecipitated with HA-tagged EcTBK1 and its truncated mutants, but not HA peptide when they were co-expressed in GS cells (Fig. 6C). Furthermore, confocal microscopic analysis showed that both EcTBK1-∆N and EcTBK1-∆C were partially co-localized with VP088 (Fig. 6D), suggesting that the interaction between VP088 and EcTBK1 was independent of its N- or C-terminal domain.

To further clarify the pathway involved in VP088-induced degradation of EcTBK1 protein *in vitro*, pFlag-VP088 and pHA-EcTBK1 were co-transfected into GS cells and treated with MG132, 3-MA, or NH_4_Cl to block different degradation pathways. Interestingly, 3-MA and NH_4_Cl, but not MG132, markedly weakened the degradation effect of VP088 on EcTBK1 (Fig. 6E through G), indicating that VP088 degraded EcTBK1 through the autophagy-lysosome pathway. Thus, we also determined whether the autophagic receptor Ecp62 was involved in the degradation process of VP088 on EcTBK1. Co-IP assays revealed that Ecp62 interacted not only with VP088 but also with EcTBK1 in co-transfected cells (Fig. 6H and I). Moreover, using Ecp62-specific siRNA, we found that the degradation effect of VP088 on EcTBK1 was markedly blocked once the protein synthesis of Ecp62 was inhibited (Fig. 6J and K). Therefore, Ecp62 was speculated to be essential for VP088-mediated autophagic degradation of EcTBK1.

### VP088 decreased EcTBK1-induced EcIRF3 phosphorylation and nuclear translocation

**Fig 7:**
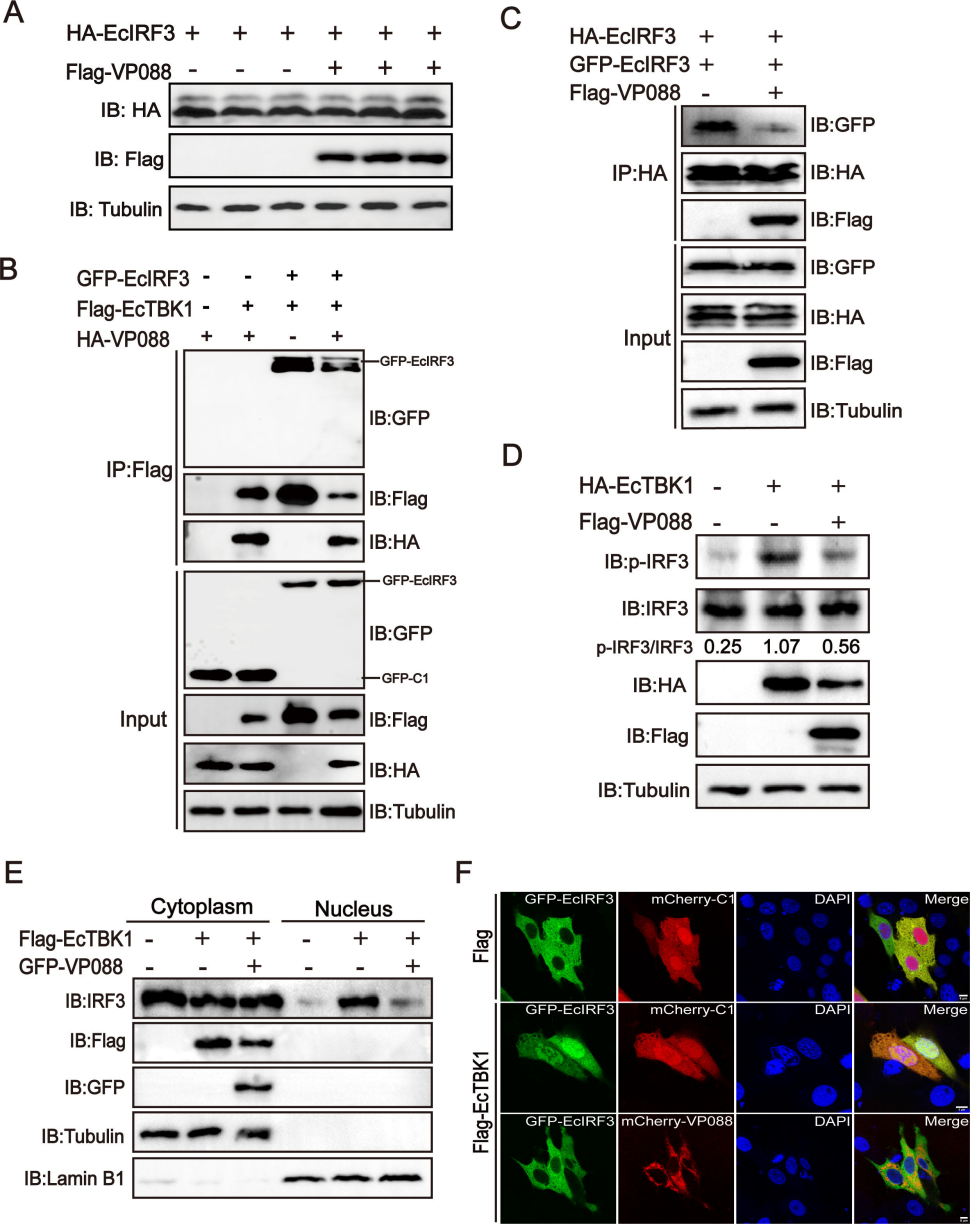
VP088 decreased EcTBK1-induced EcIRF3 phosphorylation and nuclear translocation *in vitro*. (**A**) The effect of VP088 on the stability of exogenous EcIRF3 protein in co-transfected cells. Cells were co-transfected with pHA-EcIRF3 and pFlag-VP088 and then collected for western blotting analysis. (**B**) The effect of VP088 on the formation of EcTBK1-EcIRF3 complex. Cells were transfected with pGFP-EcIRF3 and pFlag-EcTBK1 with or without pHA-VP088, and then the cells were collected for Co-IP assay and western blotting analysis. (**C**) The effect of VP088 on EcIRF3 dimerization *in vitro*. Cells were transfected with pGFP-EcIRF3 and pHA-EcIRF3 with or without pFlag-VP088 and then collected for western blotting. (**D**) The effect of VP088 on EcTBK1-induced EcIRF3 phosphorylation. GS cells were transfected with pGFP-VP088 and pFlag-EcTBK1 and then collected for detection of the phosphorylated EcIRF3 using western blotting analysis. (**E and F**) The effect of VP088 on the nuclear translocation of EcIRF3 with or without EcTBK1 by western blotting (**E**) or confocal microscopy (**F**). GS cells were transfected with pFlag-EcIRF3 and pGFP-VP088 or empty vector and then harvested for nucleocytoplasmic separation and IB analysis. In parallel, GS cells were transfected with pGFP-EcIRF3, pFlag-EcTBK1, and pmCherry-VP088 or empty vector for 48 h. Then, the cells were stained with DAPI and visualized under a confocal microscope. Scale bar, 5 µM.

Next, we also detected whether VP088 affects the stability of the transcription factor IRF3. In VP088 and EcIRF3 co-transfected cells, VP088 exhibited no obvious degradation effect on the protein abundance of EcIRF3 (Fig. 7A). However, VP088 not only markedly decreased the formation of the EcTBK1-EcIRF3 complex in co-transfected cells (Fig. 7B) but also reduced the dimerization of EcIRF3 (Fig. 7C). Moreover, VP088 markedly decreased EcTBK1-induced EcIRF3 phosphorylation (Fig. 7D). Consistently, western blotting analysis showed that VP088 markedly decreased the abundance of EcIRF3 in the nucleus fraction after EcTBK1 activation. Confocal microscopic observation also demonstrated that the fluorescence of EcIRF3 in the nucleus was markedly reduced in VP088 and EcTBK1-EcIRF3 co-transfected cells compared with EcTBK1-EcIRF3 transfected cells (Fig. 7E and F). Thus, VP088 was speculated to affect EcIRF3 activity via decreasing EcTBK1-induced EcIRF3 phosphorylation and nuclear translocation.

### VP088 attenuated the antiviral actions of EcSTING-EcTBK1-EcIRF3 axis

**Fig 8:**
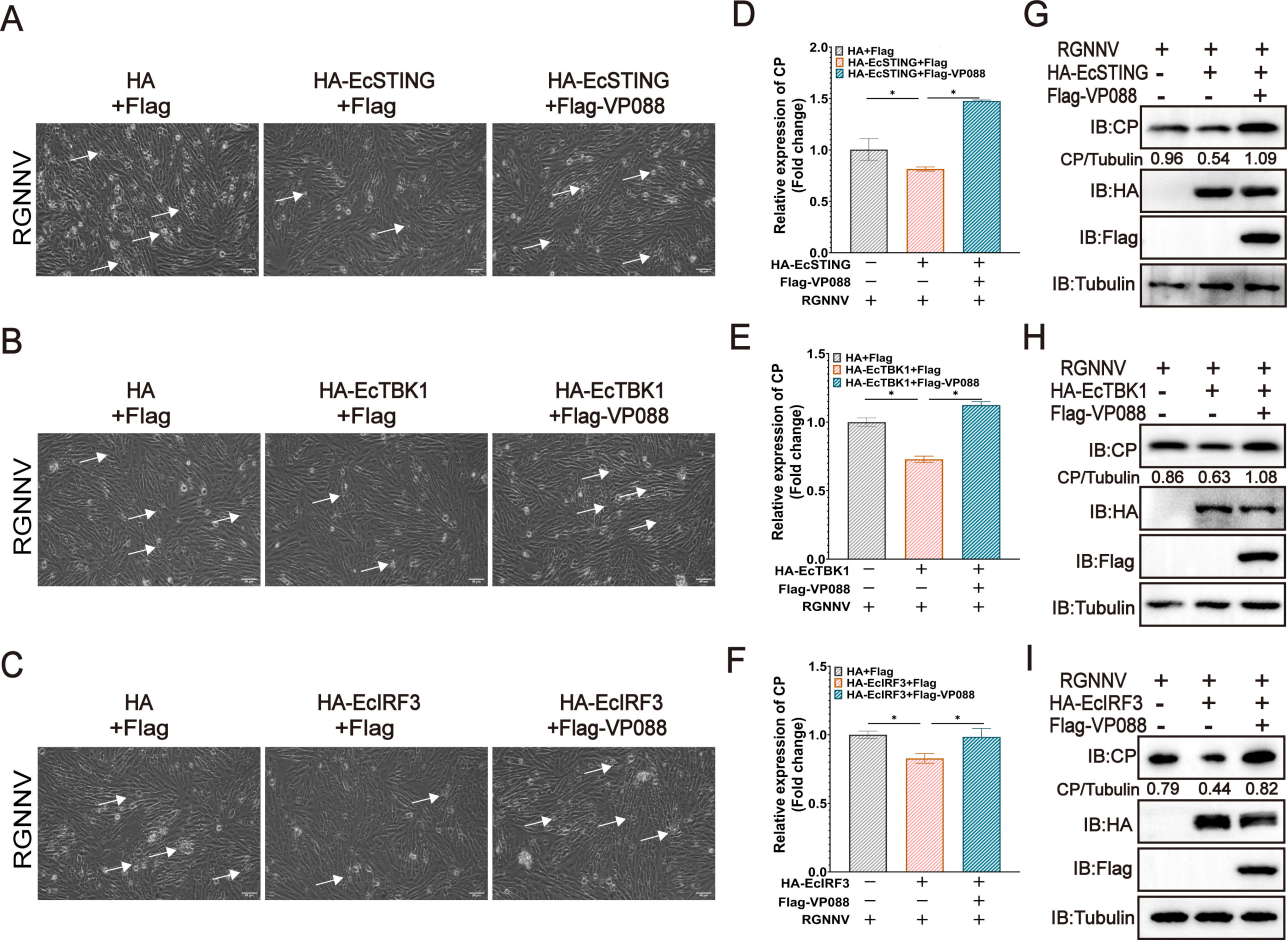
VP088 weakened the antiviral effect of EcSTING/EcTBK1/EcIRF3 upon RGNNV infection. (**A–C**) The effect of VP088 on the CPE (white arrows) progression in EcSTING (**A**), EcTBK1 (**B**), and EcIRF3 (**C**) overexpressing cells upon RGNNV infection. Cells were transfected with pHA-EcSTING, pHA-EcTBK1, pHA-EcIRF3, and pFlag-VP088 and then infected with RGNNV. The cell morphology was observed under light microscopy. Scale bar, 50 µM. (**D–F**) The effect of VP088 on the mRNA levels of RGNNV CP in EcSTING (**D**), EcTBK1 (**E**), or EcIRF3 (**F**) overexpressing cells upon RGNNV infection. Cells were transfected with indicated plasmids and infected with RGNNV as described above. The infected cells were collected for qPCR analysis. (**G–I**) The effect of VP088 on the protein synthesis of RGNNV CP in EcSTING (**G**), EcTBK1 (**H**), and EcIRF3 (**I**) overexpressing cells upon RGNNV infection. Cells were treated as described above and then collected for western blotting analysis. *P < 0.05.

Next, we also evaluated the regulatory effect of VP088 on the antiviral activities of EcSTING, EcTBK1, or EcIRF3. Overexpression of EcSTING, EcTBK1, or EcIRF3 all markedly inhibited the progression of RGNNV-induced cytopathic effect (CPE). However, their inhibitory effects were partially reversed by VP088 overexpression (Fig. 8A through C). Consistently, the transcription levels (Fig. 8D through F) and protein expression levels (Fig. 8G through I) of RGNNV CP were decreased by EcSTING, EcTBK1, or EcIRF3 overexpression but rescued by VP088 co-transfection. Thus, we speculated that VP088 antagonized the antiviral actions regulated by EcSTING-EcTBK1-EcIRF3 axis.

## DISCUSSION

During the co-evolution between viruses and their hosts, viruses have developed various strategies to evade the host immune defense. For DNA viruses, African swine fever virus (ASFV) B175L inhibited the downstream signals of IFN-mediated antiviral response by interfering with the interaction between cGAMP and STING (33). ASFV pI215L negatively regulated the cGAS-STING signaling pathway by recruiting RNF138 (34). In fish, cyprinid herpesvirus 2 (CyHV-2) KLP degraded STING by the autophagy-lysosomal pathway and further diminished the antiviral ability of STING (35). ORF67 also inhibited IFN expression by competitively obstructing STING phosphorylation (36). SGIV VP131 interacted with and degraded STING and TBK1, thereby disrupting the antiviral activity of STING or TBK1 (24). As a large DNA virus, whether more viral proteins were involved in immune evasion of SGIV remained fascinating.

Recent studies demonstrated that VP088 functioned as an endoplasmic reticulum-localized protein and participated in virus assembly (31). As an anchor protein, VP088 might recruit other viral proteins into the virus assembly sites (31). To clarify this hypothesis, we screened the potential interacting proteins using GFP pull-down assay, and the results showed that VP088 might be associated with VP018/VP068/VP156 during SGIV infection. Their interactions were verified in co-expressed cells by Co-IP analysis. Moreover, VP088 was able to alter the localization of VP018 and VP068 without SGIV infection. Interestingly, upon SGIV infection, the exogenous VP088 and VP156 were almost simultaneously transferred into the virus assembly sites, while VP018 and VP068 were translocated at the late stage of SGIV infection. Consistently, endogenous VP018, VP068, VP156, and VP088 were all present in the virus assembly sites during SGIV infection. Consistently, exogenous VP088 also colocalized with MCP (VP072) and translocated to the virus assembly sites together at the late stage of SGIV infection (31). Directional or temporal translocation events were found to facilitate localization-dependent protein interactions and contribute to either host defense or virus replication (37). Therefore, we speculated that VP088 interacted with these viral proteins in different ways and participated in virus assembly during SGIV infection. Although we also identified several cellular proteins that interacted with VP088 during infection, such as Rab7 and Rab5C, their detailed roles in SGIV replication needed further investigation.

Notably, among these interacted viral proteins, VP018 was previously elucidated to exert dual roles during SGIV infection (25, 38). VP018 not only played an important role in the expressions of viral late genes and virion assembly (38) but also abrogated EcSTING-induced IFN response via disrupting the assembly of the EcSTING-EcTBK1 and EcTBK1-EcIRF3 complexes and reducing the nuclear translocation of EcIRF3 (25). Based on our findings that VP088 prevented the trafficking of VP018 into the nucleus completely, we speculated that VP088 might also function via regulating the host IFN response. As expected, the transcription levels of IFN-related genes in VP088 alone overexpressing cells were inhibited compared with control cells, while those in VP088-silenced infected cells were significantly upregulated compared with SGIV-infected cells, suggesting that VP088 was crucial for SGIV to inhibit the IFN response. Moreover, co-transfection with VP088 significantly inhibited EccGAS-EcSTING- and EcTBK1-induced IFN response, evidenced by the decrease of the IFN promoter activities and the expression levels of IFN-related genes. Similarly, exogenous DNA and RNA-mediated IFN activation were both abrogated by CyHV-2 KLP (35). SGIV VP131 alone also weakened EcSTING-, EcTBK1-, or EcMDA5-induced IFN response (24), suggesting that VP088 might be another candidate for SGIV to evade host antiviral immune response via EcSTING-EcTBK1-EcIRF3 axis.

Although numerous viral immune evasion proteins have been reported to abrogate cGAS-STING-mediated IFN signaling, their interactions with target molecules in the cGAS-STING pathway remained at a variety of levels (39, 40). Here, the Co-IP assay showed that VP088 interacted with EccGAS, EcSTING, EcTBK1, and EcIRF3 in co-transfected cells. VP088 did not significantly alter the protein abundance of the exogenous EccGAS and EcSTING, as well as the level of EcSTING dimerization. However, VP088 reduced the assembly of the EcSTING-EcTBK1 complex. Consistently, VP088 decreased the abundance of exogenous EcTBK1 protein in a dose-dependent manner, and the degradation of EcTBK1 was via the Ecp62-mediated autophagy pathway, suggesting that EcTBK1 was a critical target for VP088 to regulate the host IFN response. Similarly, Avibirnavirus VP3 could inhibit TRAF6-mediated IFN-β production to evade host innate immunity by inducing TRAF6 autophagic degradation in a p62-dependent manner (41). Encephalomyocarditis Virus Structural Protein VP3 also triggered MAVS degradation through the p62-mediated autophagy pathway (42). Differently, Grass Carp Reovirus VP4 recruited and interacted with toll-interacting protein to degrade STING via the autophagy-lysosome pathway (10). Thus, we speculated that Ecp62-mediated autophagy was crucial for VP088-triggered EcTBK1 degradation. Whether other autophagic receptors were involved in this process needed further investigation.

As a downstream target of EcTBK1, EcIRF3 exerted the physiological function, usually accompanied by its phosphorylation, dimerization, and nuclear translocation (43, 44). The activated IRF3 subsequently caused the transcriptional activation of IFN and ISRE promoters, finally resulting in the induction of IFN-I and numerous ISGs for the establishment of an antiviral state (7). Human cytomegalovirus US9 evaded the type I interferon response by disrupting IRF3 nuclear translocation (45). HSV-1 VP24 also abrogated the interaction between TBK1 and IRF3, hence impairing IRF3 activation including its phosphorylation and dimerization (46). In our study, although the presence of VP088 showed no obvious effect on the degradation of exogenous EcIRF3 protein, this not only hindered the assembly of EcTBK1-EcIRF3 complex but also reduced EcIRF3 dimerization. Moreover, EcTBK1-activated EcIRF3 phosphorylation and nuclear translocation were also markedly decreased in VP088 co-transfected cells, thereby interfering with the activation of IFN antiviral response. Consistently, VP088 overexpression attenuated the antiviral activities of EcSTING/EcTBK1/EcIRF3 against RGNNV infection. Thus, we speculated that the attenuated activation of IRF3 was crucial for VP088 to abrogate the host IFN response.

In conclusion, our study identified the interacting components of envelope protein VP088 during virus assembly. Moreover, we for the first time demonstrated that VP088 also functioned as an immune evasion protein via Ecp62-mediated autophagic degradation of EcTBK1, thereby inhibiting the host IFN response. Our results will provide a novel molecular target for the construction of immunogenic live vaccines against grouper iridoviral diseases.

## Data Availability

All data generated or analyzed during this study have been described in the text and figures. All other data associated with this paper can be obtained from the corresponding author.

## SUPPLEMENTAL MATERIAL

Tables S1 and S2 jvi.01193-25-s0001.pdf Primers used in this study and identified proteins that interacted with VP088 during SGIV infection using LC-MS/MS.

ASM does not own the copyrights to Supplemental Material that may be linked to, or accessed through, an article. The authors have granted ASM a non-exclusive, world-wide license to publish the Supplemental Material files. Please contact the corresponding author directly for reuse.
