# Supplementary material for: SGIV envelope protein VP088 facilitated virus replication via interacting with other viral proteins and promoting p62-dependent autophagic degradation of TBK1
Source: J Virol. 2025 Dec 11;100(1):e01193-25. doi: 10.1128/jvi.01193-25 (PMC12817895; doi:10.1128/jvi.01193-25)
Supplement: Tables S1 and S2 — Primers used in this study and identified proteins that interacted with VP088 during SGIV infection using LC-MS/MS. [file jvi.01193-25-s0001.pdf]

Table S1. The primers used in this study

Primer names	Sequence (5'–3')
HA-VP088-F	CCCAAGCTTATGGGCGCAGCGCAATC
HA-VP088-R	CCGCTCGAGTCACTTTGCAGCTTCTCC
Flag-VP088-F	CCGTGGAATTCTATGGGCGCAGCGCAATCC
Flag-VP088-R	CCGACTCGAGCGTCACTTTGCAGCTTCTCCAACTTCG
GFP/mCherry-VP088-F	CCGTCTCGAGCTATGGGCGCAGCGCAATCC
GFP/mCherry-VP088-R	CCGAGAATTCGATCACTTTGCAGCTTCTCCAAC
HA-VP068-F	TACGCATCAGCGGAAATGGCGGTATATAAAAATTCCCATTTTTGGAAG
HA-VP068-R	GATATCTGCAGAATTTTGATACATTCAAAGTCCTATTTTCGGTACGT
HA-VP156-F	GATCCACTAGTCCAGTGTGGATGTCATACAAGAACGATTTACAATATC TGCTCT
HA-VP156-R	GGTTTAAACGGGCCCTCTAGATAATTTTTCAGCTGCGCGTAGG
GFP-VP068-F	TCCGGACTCAGATCTATGGCGGTATATAAAAATTCCCATTTTTGGAAG
GFP-VP068-R	AGATCCGGTGGATCCTGATACATTCAAAGTCCTATTTTCGGTACGT
GFP-VP156-F	CTAGCGCTACCGGACTCAGAATGTCATACAAGAACGATTTACAATATC TGCTCT
GFP-VP156-R	GCGATGGATCCCGGGCCCGCATAATTTTTCAGCTGCGCGTAGG
HA-Ecp62-F	CGCCTCGGATCCATGTCGGTGACGGTGAAAGC
HA-Ecp62-R	CCGAGAATTCCATCATGGCTGGTGTGGGCGG
β-Actin-F	TACGAGCTGCCTGACGGACA
β-Actin-R	GGCTGTGATCTCCTTCTGCA
si-VP088-1-sense	GUGUAAAGAUUGUCACAAA
si-VP088-2-sense	AGGACGAACUGAUUGACAA
si-VP088-3-sense	CAGCGACAUCGUUUCUCAA
si-Ecp62-1-sense	GAAGGAUGAGUCGGUGAAATT
si-Ecp62-2-sense	GUGGAUGAAGUGGAUGAGATT
si-Ecp62-3-sense	GCUGUCGAUGGGAUUCACUTT

Table S2. Identified proteins interacted with VP088 during SGIV infection using LC-MS/MS

Accession ID	Protein name	MW(kDa)	Best-matched Peptide
AAS18171.1	SGIV VP156	31.22	GGGSEEITFLK
AAS18033.1	SGIV VP018	32.70	AGVGVVWGPEER
AAS18083.1	SGIV VP068	29.82	LVASGGVDGIK
AAS18087.1	SGIV VP072 (major capsid protein)	50.78	SASLTYENTTR
XP_049446734.1	heat shock protein HSP 90-beta isoform X1	83.8	ELISNASDALDKIR
XP_033471330.1	ATP synthase subunit beta, mitochondrial	55.18	FTQAGSEVSALLGR
H9BW96.1	Ras-related protein rab7	23.84	LVTMQIWDTAGQER
XP_033504966.1	phosphoglycerate kinase 1	44.89	LGDVYVNDAFGTAHR
QOE32863.1	ras-related protein Rab-5C	23.78	FEIWDTAGQER
XP_033495290.1	V-type proton ATPase catalytic subunit A	68.68	TALVANTSNMPVAAR
